# Quadruple Disruption of the Superior Shoulder Suspensory Complex: A Case Report on Surgical Intervention Following High-Energy Trauma in a Professional Cycling Athlete

**DOI:** 10.7759/cureus.80451

**Published:** 2025-03-12

**Authors:** Maiwand Sarwari, Anne Ottenhof, Sjoerd H den Uil, Jelle J Posthuma

**Affiliations:** 1 Department of Surgery, Flevoziekenhuis, Almere, NLD; 2 Department of Surgery, Amsterdam University Medical Center, Amsterdam, NLD

**Keywords:** complex shoulder girdle injuries, high-energy trauma, quadruple disruption, sssc, superior shoulder suspensory complex

## Abstract

The superior shoulder suspensory complex (SSSC), which consists of several anatomical structures, plays a crucial role in stabilizing the shoulder girdle. While single disruptions can often be managed non-operatively, double disruptions typically require surgical intervention due to increased risks of complications like persisting instability, malunion, and/or non-union. In addition, quadruple disruptions are extremely rare and usually caused by high-energy trauma. Surgical repair is required to optimize functional outcomes.

We present the case of a 34-year-old male professional cyclist who sustained a quadruple disruption of the SSSC after a high-speed fall. Computed tomography (CT) imaging revealed fractures of the distal clavicle, acromion, coracoid, and glenoid. In addition, the rider sustained an olecranon fracture. Given the high likelihood of persistent instability with non-surgical treatment, surgical fixation of the distal clavicle, acromion, and coracoid was performed, alongside olecranon stabilization. Postoperatively, the patient regained full function within three months and returned to competitive cycling.

This case illustrates the rare occurrence of quadruple disruptions of the SSSC following high-energy trauma and the need for surgical intervention. The patient achieved excellent functional outcomes. This highlights the importance of timely surgical intervention to prevent complications and support full recovery.

## Introduction

The shoulder girdle’s stability is maintained by two interconnected arches, forming a structure known as the superior shoulder suspensory complex (SSSC) [1]. The SSSC is a ligamentous structure consisting of a ring of anatomical structures, including the glenoid process, distal clavicle, acromioclavicular joint, acromial process, coracoid process, and coracoclavicular ligaments. Single disruptions of the SSSC are relatively common and often managed non-operatively in the majority of cases. In contrast, double disruptions occur less frequently and typically necessitate surgical intervention due to the heightened risk of complications, such as persistent instability, pain, malunion, and/or non-union when treated non-operatively. Triple and, especially, quadruple disruptions of the SSSC are extremely rare, with only a few cases of a quadruple disruption documented in the literature [2-5]. Given that quadruple disruption of the SSSC causes severe instability, surgical intervention is generally recommended to achieve anatomical reduction and optimize functional outcomes. This case report details a 34-year-old male patient who sustained a quadruple disruption of the SSSC following high-energy trauma during a cycling event, involving fractures of the distal clavicle, acromion, coracoid, and glenoid, which were subsequently managed operatively.

## Case presentation

**Figure 1:**
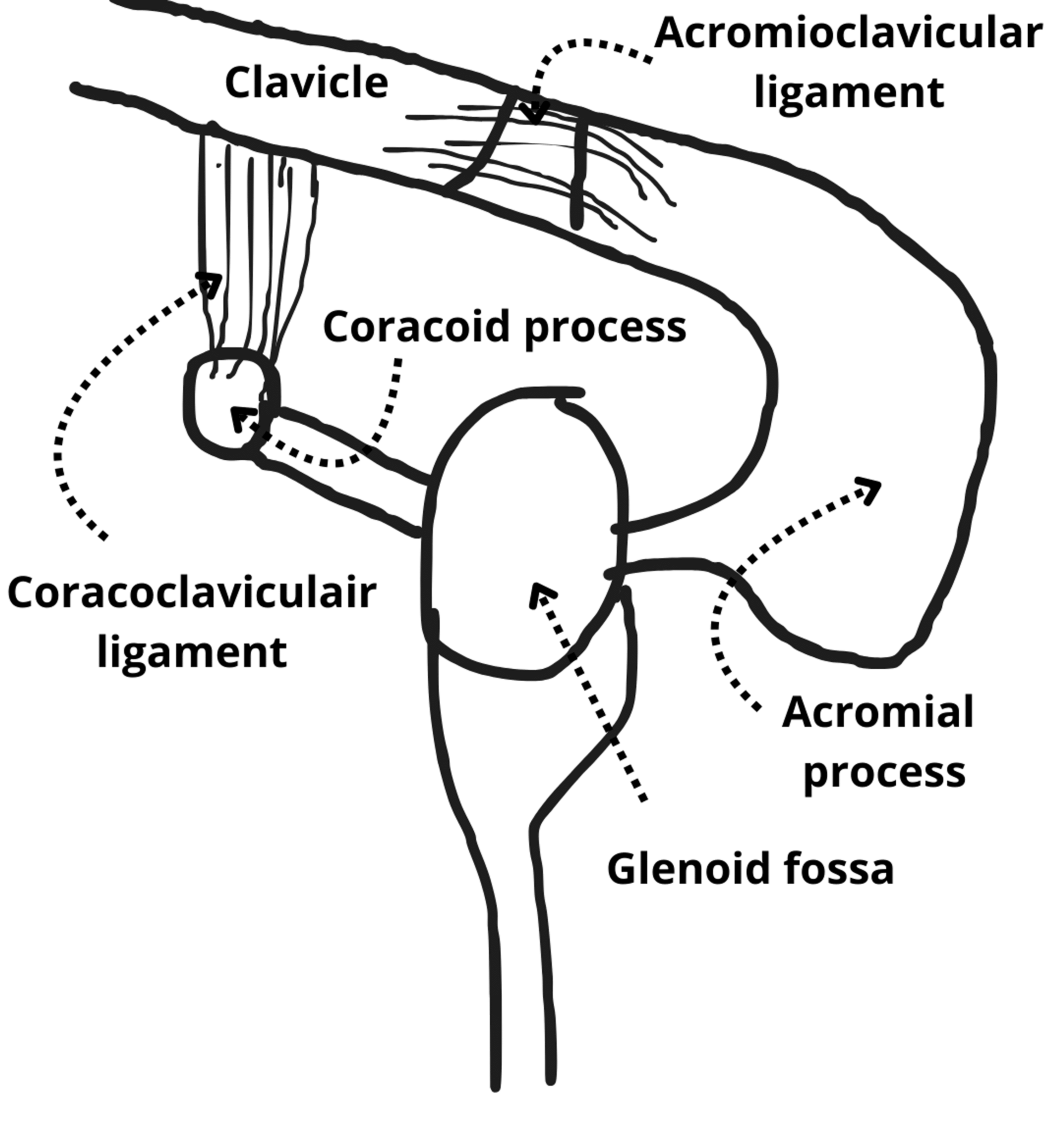
Schematic representation of the SSSC SSSC, superior shoulder suspensory complex Image credits: The authors of this article

A 34-year-old Caucasian male professional cyclist presented with a left shoulder injury after a fall while cycling at a speed of 45-50 kilometres per hour (km/h). The patient had no remarkable medical history, no known allergies, and was not on any medication. The patient presented with complaints of restricted range of motion of the left shoulder. Physical examination showed restricted motion of the left shoulder with superficial abrasions without neurovascular damage. As previously mentioned, the SSSC consists of a ring of structures in the shoulder, which initially raised a strong suspicion of injury to one or more structures within the SSSC (Figure 1). 

**Figure 2:**
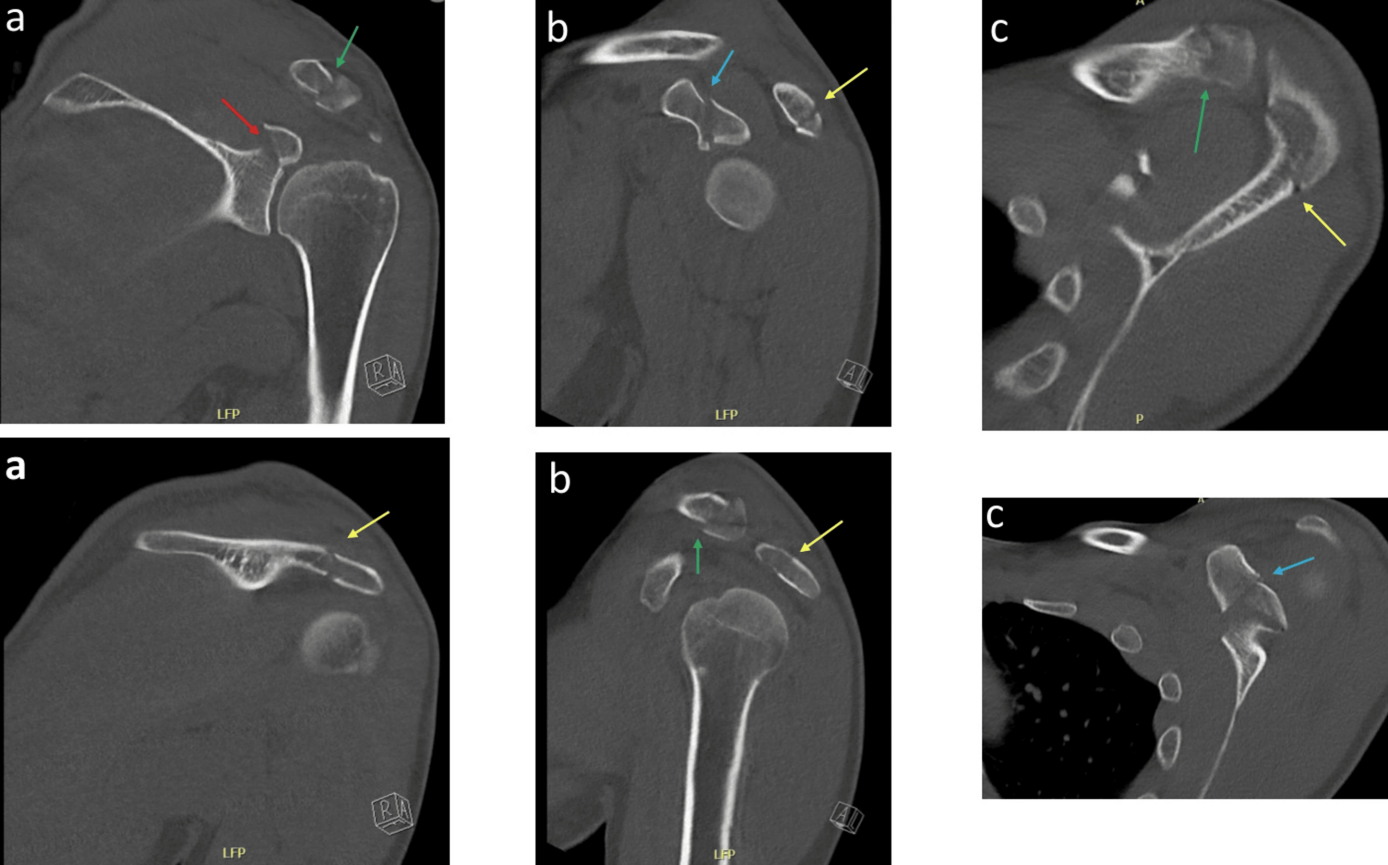
Preoperative computed tomography (CT) images of the shoulder girdle Preoperative CT images in the coronal (a), sagittal (b), and axial (c) planes. The arrows indicate fractures of the glenoid (red), acromion (yellow), distal clavicle (green), and coracoid (blue).

**Figure 3:**
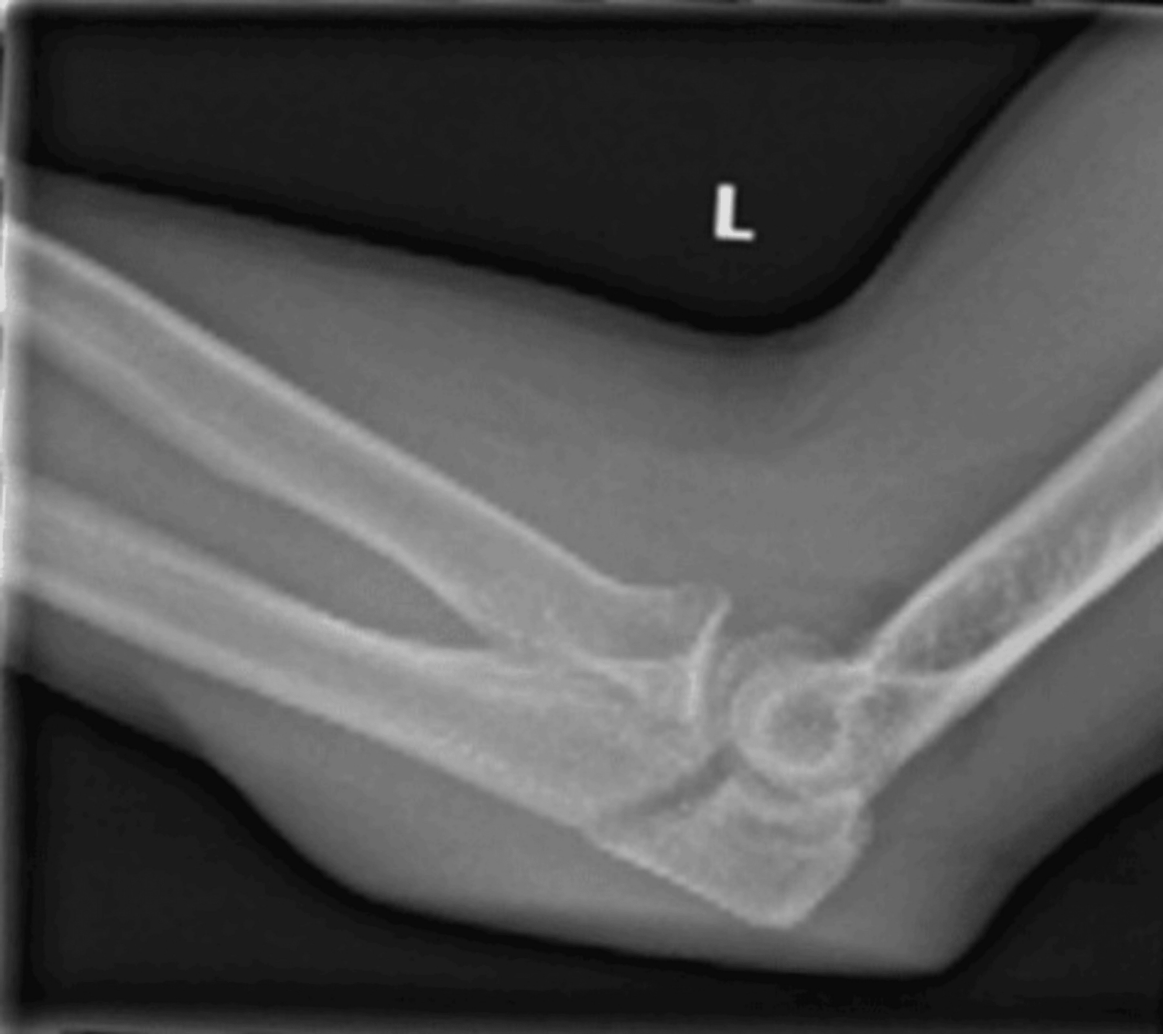
Preoperative X-ray of the olecranon Preoperative X-ray in the sagittal view of the olecranon fracture.

Initial computed tomography (CT) imaging revealed a distal clavicle fracture, a glenoid fracture (Ideberg type 3), an acromion fracture, and a coracoid fracture (Ogawa type 2) (Figure 2). In addition, he sustained a left olecranon fracture (Figure 3). After initial conservative management, the patient was referred to our clinic five days after sustaining the injury, specialized in managing sport-related trauma. Given the quadruple disruption of the SSSC, surgical intervention was necessary. Based on the available literature, although limited, at least two components should be fixated to optimize shoulder stabilization. The exact two components that should be fixated are not entirely clear, as the choice depends on the degree of displacement of the fracture. In shared decision making with the patient, operative management was chosen, and, in this case, the distal clavicle, the acromion process, and the coracoid process were fixated, as described below. The glenoid was not fixated due to the minimal displacement of the fracture. This procedure was combined with simultaneous fixation of the ipsilateral olecranon fracture.

**Figure 4:**
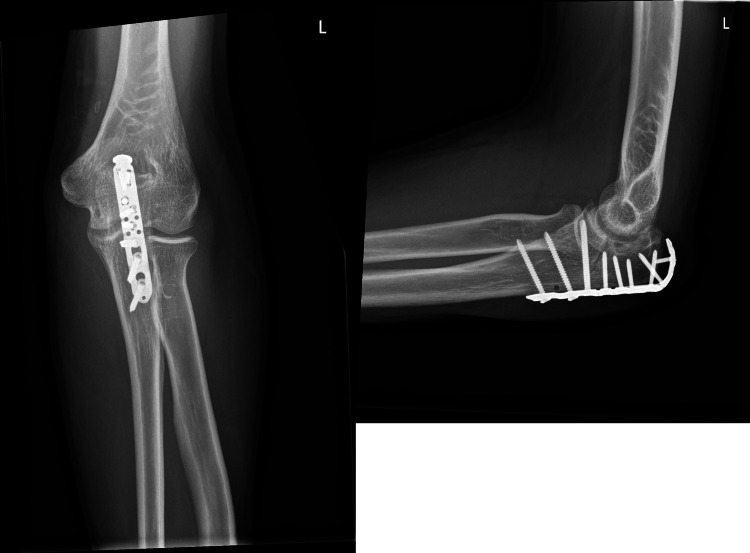
Six-week postoperative X-ray of the olecranon A six-week postoperative X-ray in the coronal (a) and sagittal (b) views of the fixed olecranon demonstrating progressive bone healing and proper alignment of the elbow.

The patient was positioned in the beach chair position. At first, the olecranon was fixed. A curvilinear incision was made over the olecranon, and the fracture underwent nettoyage and reduction before applying a variable angle (VA) locking-compression olecranon plate (Figure 4).

**Figure 5:**
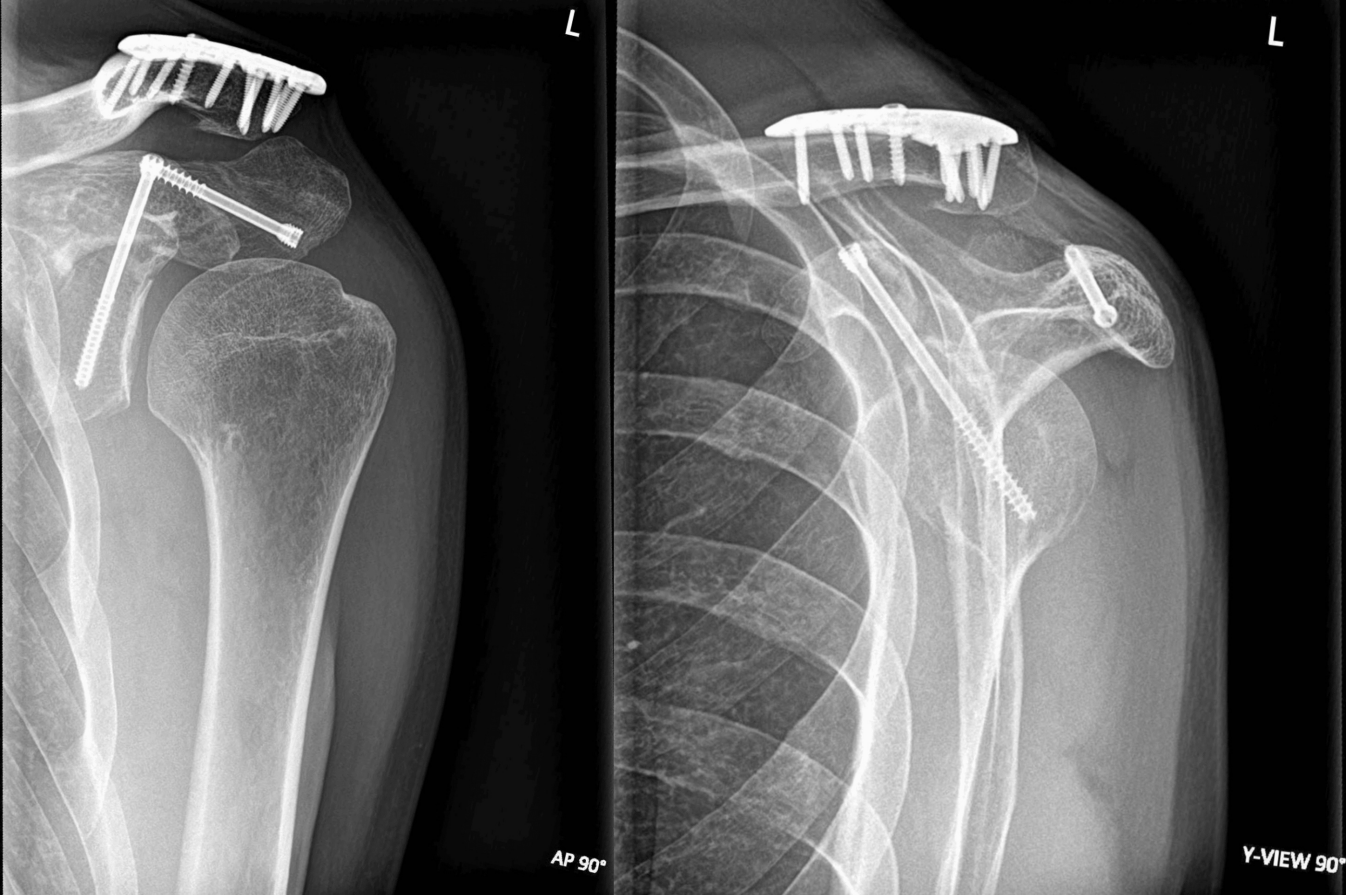
Six-week postoperative radiograph of the shoulder girdle A six-week postoperative radiograph in the coronal view demonstrating progressive bone healing in the shoulder.

Then we continued with stabilization of the shoulder. A skin incision was made over the lateral clavicle. The fracture was reduced and temporarily stabilized with a Kirschner-wire (K-wire). A VA lateral clavicle plate was then positioned and fixated using 2.7 mm locking screws, and a 3.5 mm cortical screw was placed medially. Perioperative radiograph confirmed adequate alignment and fixation. Next, a small incision was made to access the tip of the coracoid process. A K-wire was inserted at the tip of the coracoid process and used to reduce the fracture. Subsequently, an additional K-wire for a 4.5 headless compression screw (HCS) was inserted under fluoroscopy. Hereafter, the HCS was inserted with satisfactory grip and fixation. Finally, an additional 4.5 mm HCS screw was placed to fixate at the acromial fracture, in a lateral to medial direction using a small stab-incision (Figure 5).

The final perioperative radiological evaluation showed satisfactory results for both injuries. The wound was thoroughly irrigated and closed with a resorbable monofilament suture.

A staged mobilization protocol with specific movement restrictions was implemented postoperatively to optimize the patient’s recovery. During the first two weeks after surgery, the shoulder was immobilized with a sling, while elbow flexion and extension exercises were allowed. After two weeks, the abduction and anteflexion movements were allowed to 90°, until six weeks postoperatively.

**Figure 6:**
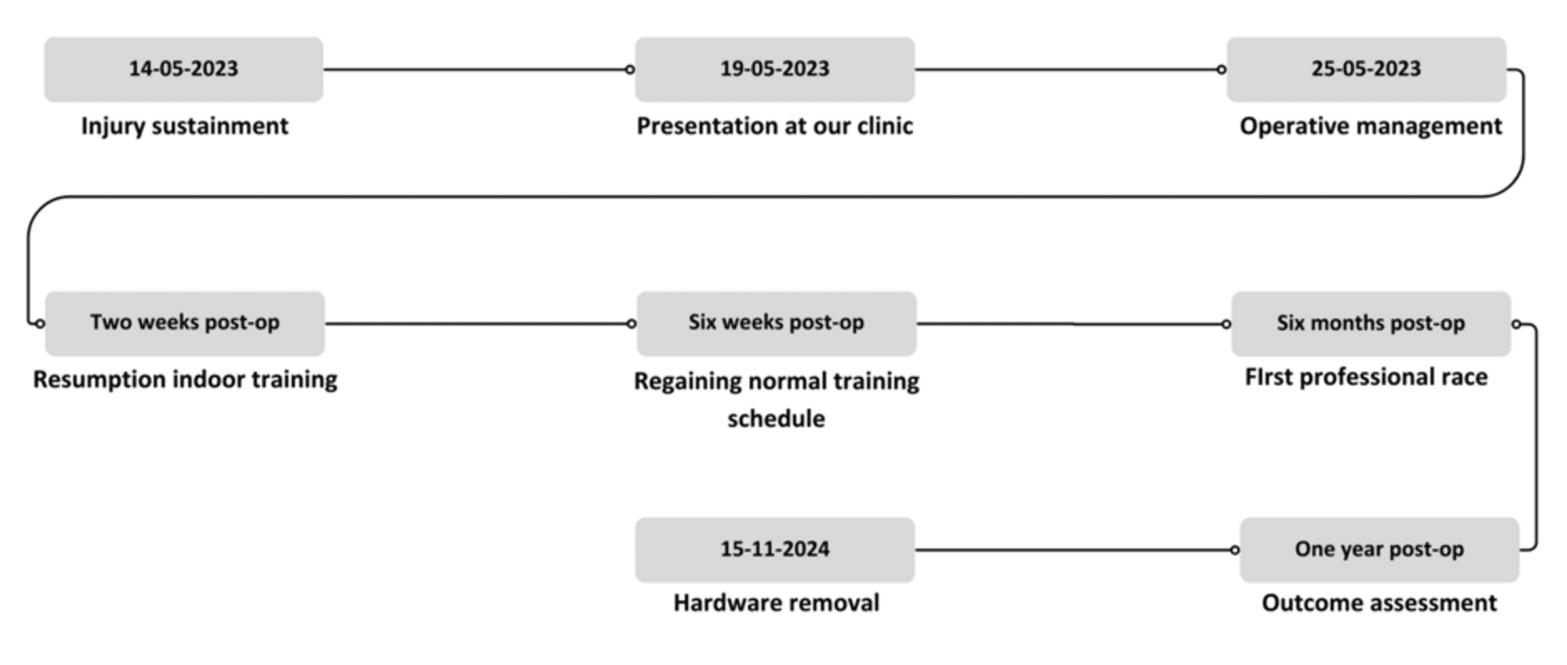
Graphical timeline of clinical course and outcomes

Postoperatively, the patient recovered remarkably quickly. Two weeks after the surgical procedure, he was able to resume indoor training. By six weeks, he had regained his normal training schedule and successfully competed in his first race six months postoperatively. Despite maintaining an elbow extension limitation of approximately 10°, his one-year postoperative outcomes were excellent. The patient achieved the following scores on functional outcome assessments: 95/100 on the Kerlan-Jobe Orthopaedic questionnaire [6], 100/100 on the Constant-Murley score [7], 0.83 on the Disabilities of the Arm, Shoulder, and Hand (DASH) questionnaire [8], and 91.67/100 on the Oxford Elbow Score averaged across the three domains [9]. Due to restricted extension and pain while leaning on the elbow, the patient underwent removal of the olecranon hardware 20 months postoperatively. Following the procedure, the elbow now exhibits a 2° extension limitation, improved from the original 10° restriction. This alleviated the patient’s discomfort. Additionally, the patient experienced discomfort while wearing a backpack on the left shoulder, prompting the removal of clavicular hardware simultaneously. This resulted in relief of discomfort, with no postoperative complications (Figure 6).

## Discussion

Isolated disruptions of the SSSC are often amenable to conservative treatment, typically yielding good functional outcomes. However, double, triple, or quadruple disruptions present a more complex scenario, usually necessitating surgical intervention due to a heightened risk of persistent instability, which can lead to pain, reduced function, malunion, and/or non-union. In our case, the patient was initially treated conservatively for the duration of five days; however, due to persisting pain and reduced function, the patient was referred to our clinic, specialized in managing sport-related trauma.

In this case, the patient sustained multiple fractures, highlighting the potential for misdiagnosis in cases with severe shoulder girdle injuries. While isolated fractures to the clavicle, acromion, glenoid, or coracoid can often be managed conservatively, the combination of these fractures necessitated surgical fixation to ensure adequate stability and function of the shoulder. In addition, similar to the pelvis, the shoulder girdle has a “ring,” and displacement in one segment frequently suggests the possibility of associated injuries in other components of the ring [10]. This interconnectedness within the SSSC highlights the importance of surgically addressing multiple fractures to maintain proper stability and function. Therefore, we strongly recommend that if a fracture is identified in any component of the SSSC following high-energy trauma, clinicians should also assess for fractures in other components. This is to avoid underdiagnosis of multiple SSSC disruptions.

The mechanism of injury in this case, a high-speed fall during cycling, aligns with trends documented in the literature. For example, Lecoq et al. reported a case following a traffic accident [11], and Jung et al. described a case resulting from a high-altitude fall [12]. These prior cases support the hypothesis that high-energy trauma frequently results in one or multiple disruptions of the SSSC. Their findings support our surgical strategy, emphasizing that a timely intervention is critical to avoid complications associated with conservative management. In case of triple disruptions, surgical stabilization is essential for optimal recovery. Oh et al. make a compelling argument for early surgical intervention, reporting significantly improved functional outcomes when key components of the SSSC are addressed [13]. Guided by this evidence, we opted for surgical fixation, especially considering the patient’s profession as a professional cyclist, where a timely return to competition and optimal shoulder function was essential. Therefore, we fixated the distal clavicle, acromion, and coracoid to restore stability and prevent potential instability-related complications. A possible complication of this procedure was the need for removal of the olecranon and clavicle hardware due to discomfort. However, the hardware removal resulted in relief of discomfort and an improvement of 8° in the previously reported extension limitation of the olecranon.

## Conclusions

This case report highlights a rare occurrence of quadruple disruptions of the SSSC following high-energy trauma in a high-performance athlete. In addition, this case highlights the fact that sport-related trauma can result in specific fracture patterns that require careful diagnosis and treatment by experienced physicians based upon the needs of high-performance athletes. In our case, minimally invasive repair resulted in excellent postoperative functional outcomes, allowing the patient to return to competition within six months.
